# Smoothing Complete Feature Pyramid Networks for Roll Mark Detection of Steel Strips

**DOI:** 10.3390/s21217264

**Published:** 2021-10-31

**Authors:** Qiwu Luo, Weiqiang Jiang, Jiaojiao Su, Jiaqiu Ai, Chunhua Yang

**Affiliations:** 1 School of Automation, Central South University, Changsha 430006, China; luoqiwu@csu.edu.cn (Q.L.); irvingjwq@csu.edu.cn (W.J.); sujiaojiao@csu.edu.cn (J.S.); ychh@csu.edu.cn (C.Y.); 2 School of Computer Science and Information Engineering, Hefei University of Technology, Hefei 230009, China

**Keywords:** surface defect detection, roll marks, hot-rolled steel, feature pyramid networks (FPN)

## Abstract

Steel strip acts as a fundamental material for the steel industry. Surface defects threaten the steel quality and cause substantial economic and reputation losses. Roll marks, always occurring periodically in a large area, are put on the top of the list of the most serious defects by steel mills. Essentially, the online roll mark detection is a tiny target inspection task in high-resolution images captured under harsh environment. In this paper, a novel method—namely, Smoothing Complete Feature Pyramid Networks (SCFPN)—is proposed for the above focused task. In particular, the concept of complete intersection over union (CIoU) is applied in feature pyramid networks to obtain faster fitting speed and higher prediction accuracy by suppressing the vanishing gradient in training process. Furthermore, label smoothing is employed to promote the generalization ability of model. In view of lack of public surface image database of steel strips, a raw defect database of hot-rolled steel strip surface, CSU_STEEL, is opened for the first time. Experiments on two public databases (DeepPCB and NEU) and one fresh texture database (CSU_STEEL) indicate that our SCFPN yields more competitive results than several prestigious networks—including Faster R-CNN, SSD, YOLOv3, YOLOv4, FPN, DIN, DDN, and CFPN.

## 1. Introduction

As one of the most important fundamental materials in steel and iron industry, steel strips are extensively used in automobile manufacturing, locomotives, aerospace, precision instrumentation, etc. For thin and wide flat steel, surface defects are the greatest threat to the product quality. Even for occasional internal defects, morphological changes will arise on the surface with a large probability. Any quality problems suffering on steel surface would give rise to irretrievable economic and reputation losses to both the steel company and end use customer. To cope with the above issue, automated visual inspection (AVI) instrument targeting on surface quality emerges as a standard configuration for flat steel mills [1].

Among the numerous categories of surface defects of hot-rolled steel strips, the roll mark is put on the top list of the most serious defects by steel mills. As a typical representative of roll marks, the roller cracking defect makes the surface extremely uneven. After the downstream continuous rolling process, these defects would transform into bumps or even holes. What is worse, such defects often occur to periodical and continuous distribution. To sum up, roll mark is one of the most harmful defects threatening steel surface quantity. Consequently, how to rapidly and accurately detect roll marks is significant for the surface defect AVI instrument.

**Figure 1:**
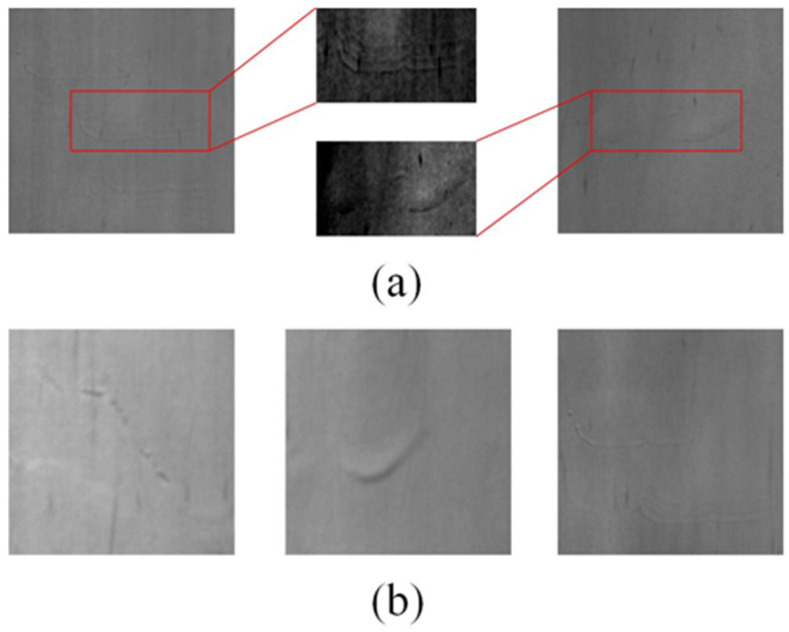
Characteristics of roll marks. (**a**) Two samples of roll marks, red rectangles mark the roll marks which has low contrast with the background and we make the roll marks explicit; and (**b**) three roll marks with completely distinct appearances.

To be specific, roll marks have two characteristics as shown below: (1) Low contrast: Roll marks manifest with concave and convex manner, however, their deformation is very shallow. Thus, as shown in Figure 1a, roll marks usually show very low contrast with the background. (2) Large intra-class distance: The appearances of roll marks are diverse, irregular, and multiscale. Figure 1b shows three patches of roll marks with completely distinct appearances. Besides, massive pseudo defects, random noises, and aperiodic vibration degrade surface image quality of steel strips under the harsh industrial environment of hot-rolling line. In other side, the fine resolution requirement of defects and high rolling speed enforce the camera device to constantly generate massive image data. To sum up, the online roll mark detection in this paper is essentially a tiny target inspection task in high-resolution images captured under harsh environment.

The conventional steel surface defect detection method based on computer vision usually be spilt into three classes: conventional statistical, spectral and model-based. Neogi et al. [2] proposed a global adaptive percentile thresholding scheme based on gradient images to separate defect selectively. It can precisely retain the defect edges regardless of the scales of defects. As a classical operator, local binary pattern (LBP) is extensively used to characterize local texture features of images, which benefit from its rotation invariance and gray invariance [3]. Song et al. [4] designed an AECLBP that regarded the surrounding gray values as its central gray value. AECLBP had achieved 98.93% accuracy on NEU datasets and great robustness to noise. Luo et al. [5] proposed a GCLBP by first exploiting the non-uniform patterns information to enrich the descriptive information, GCLBP achieved 99.11% accuracy on NEU datasets. However, the conventional statistical methods have the following weaknesses: large computation requirement, unsatisfactory real-time performance, scale sensitivity, and noise sensitivity [6]. Song et al. [7] adopted wavelet transform to construct a scattering convolution network (SCN) which can enhance the tolerance ability of local and linearized deformations, and SCN obtained accuracy of 97.22% on hot-rolled steel strip defect detection application. Nonetheless, the spectral methods have the following weaknesses: they are easily affected by feature correlations between the scales, and high computation and memory requirements [8]. Xu et al. [9] designed a hidden Markov tree model called CAHMT based on an assertion that the correlation of wavelet coefficients of flat steel surface images at different scales satisfies Markov property. CAHMT’s detection false rate is as low as 3.7%. Fofi et al. [10] designed a non-parametric texture defect detection method by using Weibull features. It performs well on DAGM database. However, it is hard for Weibull distribution to handle defects with gradual intensity or with low contrast. Hence, Liu et al. [11] proposed a Haar–Weibull-variance (HWV) model by using Haar features from local patches. This method achieved accuracy of 96.2% on a hot-rolled steel surface defect dataset. Nonetheless, the above model-based methods have the following disadvantages: spatial limitations and failure to detect tiny defects among global images. In conclusion, traditional computer vision methods had achieved ideal results in the steel surface defect detection application. However, the aforementioned traditional methods only take low-level features into account, which could not fully characterize the image features and semantic details. It is also noteworthy that there are also nearly no specific roll mark detection methods and datasets.

For the past few years, hardware computing devices boosting and the continuous expansion of public datasets have facilitated the development of neural networks [12]. Object detection networks based on neural networks are theoretically divided into two branches, namely one-stage detection and two-stage detection. One-stage detection networks operate feature extraction and prediction regression in an integral network orderly. Li et al. [13] made the YOLO all convolutional to detect flat steel surface defects and reached accuracy of 99% with 83 FPS. Liu et al. [14] improved SSD network and put forward RAF-SSD, which obtained 75.1% mAP in NEU database. However, the above methods failed to cope with the multiscale defects and tiny defects. Two-stage detection networks firstly propose a certain amount of proposal boxes, then classify them through another convolutional neural network. Dong et al. [15] proposed a pyramid feature fusion and global context attention network for pixel-wise detection of surface defect, called PGA-Net, which achieved 82.15% mean pixel accuracy in NEU-Seg. Cha et al. [16] designed a structural visual inspection method to decrease the processing time of Faster R-CNN which be capable of detect multiple classes defects. The ointment is that the real-time performance of Faster R-CNN is not satisfactory. He et al. [17] proposed a multi-scale feature fusion network (MFN), which obtained 82.3% mAP in NEU-Det. Song et al. [18] proposed a novel encoder–decoder residual network (EDRNet), which can accurately segment the whole defect instances with clear-cut boundary and effectively filter out irrelevant background noise. Nonetheless, the excellent performance of aforementioned deep learning methods was processed in image patches, such as NEU-Det (1200 samples with the resolution of 200 × 200), which is widely used among peers. These samples were beforehand processed by aforehand detection, selection and segmentation based on prior knowledge, which are rather easier to handle than our target (detect tiny defects in high resolution images). On the realistic industrial production line; however, the images acquired by the steel defect detection system were wide and high-resolution [19]. For example, the image resolution of the surface defect detection system developed by our research team in the early stage is 1024 × 4096. In consideration of the lack of public databases for steel strip surface defect inspection field, we open a raw defect database of hot-rolled steel strip surface CSU_STEEL for the first time, which contains six kinds of defects including roll mark, elastic deformation, wave, inclusions, oxide scale, and scratches with 1024 × 4096 resolution. *As far as we know, this dataset is the first wide-format high-resolution hot-rolled steel strip surface original image dataset.* Faced with the challenge of tiny target detection in high resolution image capture under harsh environments, a novel method—namely, smoothing complete feature pyramid networks (SCFPN)—is proposed for the above focused task. The concept of complete intersection over union (CIoU) is applied in feature pyramid networks to obtain faster regression speed and higher prediction accuracy by suppressing vanishing gradient in training process. In addition, label smoothing is employed to improve the generalization ability of model.

The rest of this paper is organized as follows. Section 2 elaborates the proposed SCFPN in detail. Section 3 will introduce our experiments setting. Afterwards, our experiments are evaluated quantitatively and qualitatively, and the experimental results on defect detection will be analyzed in Section 4. Section 5 will discuss the results. Finally, Section 6 will conclude this paper and discuss the future work.2. Materials and Methods

**Figure 2:**
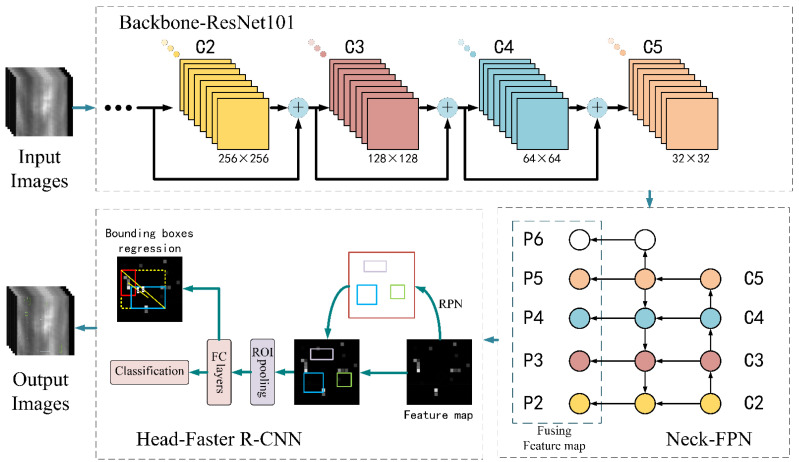
Structure of smoothing complete feature pyramid networks (SCFPN). Backbone: ResNet101, Neck: FPN, Head: Faster R-CNN, Bounding boxes regression: CIoU loss, Classification: Cross-entropy loss with label smoothing.

This paper concentrates on the steel strip surface roll mark detection problem, A targeted two-stage object detection method—namely, smoothing complete feature pyramid networks (SCFPN)—is designed, and the structure of SCFPN is shown in Figure 2. Primarily, the backbone extracts feature of multi-levels from input images. ResNets increase layers of networks without causing degradation problem. The deeper networks can extract more abstract feature with robust semantic information. Feature maps range from bottom (fine resolution) to top (coarse resolution) in the pyramid hierarchy are utilized to construct feature pyramid (Neck) by aggregations between fusion of multi-scale features. SCFPN only acquires a single-scale image of an arbitrary size, and builds feature pyramid at multiple scales by convolution. Afterwards, Faster R-CNN (Head) is applied to execute bounding boxes regression and classification tasks. Concretely, loss function of bounding boxes regression uses complete intersection over union (CIoU) loss which provides faster fitting speed and higher prediction accuracy. Loss function of classification is the Cross-entropy loss with label smoothing which is employed to enhance the generalization ability of the model. After the above steps, the networks export output images with predicted boxes and labels [20].

## 2. Materials and Methods

### 2.1. Feature Pyramid Networks

Feature pyramid networks (FPN) adopt pyramid hierarchy to collect feature information from low-level to high-level. In detail, a pair of multi-scale feature pyramids are identically constructed by upsampling and downsampling, so as to fuse features with low resolution, abundant semantic information and features with fine resolution, inferior semantic information through top-down pathway and lateral connections.

#### 2.1.1. Bottom-Up Pathway

The bottom-up pathway is constructed by the feedforward computation of the backbone ResNets, which generated multiscale feature maps with proportion of 2. We call those layers which possess feature maps of same size as different stage respectively. The final layer of each stage has the most abundant features, so we select the final layer of each stage as feature maps to construct feature pyramid. Concretely, for ResNets101, we named the output of last residual blocks as {C2, C3, C4, C5}, and which have receptive field of {4 × 4, 8 × 8, 16 × 6, 32 × 32} pixels severally [21].

#### 2.1.2. Top-Down Pathway and Lateral Connections

**Figure 3:**
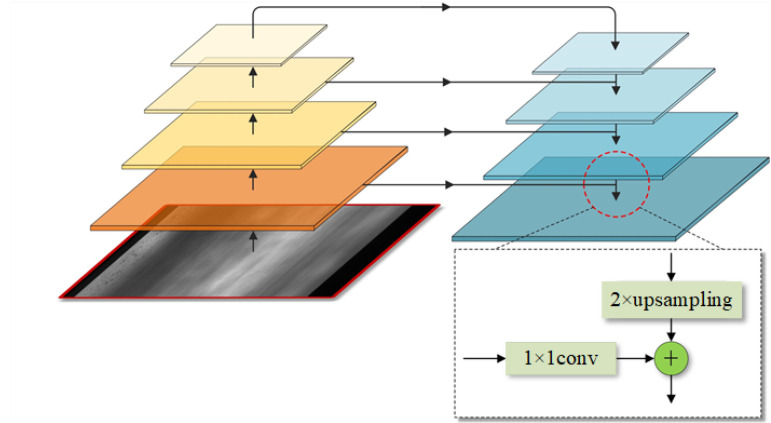
Structure of feature pyramid. Orange feature pyramid is constructed by the feedforward computation of the backbone (the bottom-up pathway), blue feature pyramid is merged by top-down pathway and lateral connections. “+” indicates pixel-level addition.

The top-down pathway upsamples (2 × upsampling) feature maps which are robust in semantic information, but with lower resolution to produce finer resolution features. In order to acquire features with robustness, lateral aggregations are used to fuse feature levels of the same size spatially from the bottom-up pathway pyramid and top-down pathway pyramid. The features in the bottom-up pathway are poor in semantic information expression, but their information of location is more accurate because they were produced by input images originally. Figure 3 shows the structure of feature pyramid. The way of lateral aggregations is a pixel-level addition. A new feature pyramid is set up after repeating the above operation, which has same structure of the previous pyramid but is robust semantically. As shown in Figure 2, the convolutional level C6 is construct by simply 1 × 1 conv from C5, which has the stronger sematic information and minimum resolution. Then, the final feature maps are generated by 3 × 3 convolution on each merged map. we name these merged feature maps as {P2, P3, P4, P5, P6}} which possess same resolution as {C2, C3, C4, C5, C6} severally.

We apply shared classifiers/regressors among all levels of the feature pyramid, and each level has d dimension (we set d as 256) similarly. Thus, all extra convolutional layers have 256-channel outputs. Even more, these extra layers do not possess non-linearities.

#### 2.1.3. Feature Pyramid Networks for RPN

RPN (Region Proposal Network) is a sliding-window class-agnostic object detector [22]. So as to employ RPN in FPN, the below modification should be adapted. In FPN, RPN are adapted to each level on feature pyramid and attach a head (3 × 3 conv and two sibling 1 × 1 conv) respectively, operating object/non-object binary classification and bounding box regression. Different level will equip relevant anchors with different scales. The feature level {P2, P3, P4, P5, P6} have anchors of {32^2^, 64^2^, 128^2^, 256^2^, 512^2^} pixels r severally. Anchors from each level has three aspect ratios of {1:2, 1:1, 2:1} respectively. In accordance with the classification rules, if a proposal box has the largest IoU with the ground-truth box or has IoU greater than threshold value (say, 0.69), it will be regarded as a positive label. While proposal boxes have IoU with any ground-truth boxes which below threshold value (say, 0.32) will be regarded as negative label. We also share the params of the heads among arbitrarily feature pyramid levels in order to maintain the good performance of sharing parameters (levels share similar semantic levels).

#### 2.1.4. Feature Pyramid Networks for Faster R-CNN

Faster R-CNN is usually applied as detection head which uses Region-of-Interest (RoI) pooling to extract features. In order to use Faster R-CNN in FPN, similarly, we need to assign RoIs of different scales to each level of feature pyramid. We imitate the allocation method strategy of RoI proposal in image pyramids. Theoretically, the equation to assign a RoI to the level Pk of feature pyramid can be expressed as

(1) $$k=[k_{0}+\log_{2}\left(\sqrt{wh}/224\right)]$$

where *w* indicates the width of an RoI, *h* indicates the height of an RoI, 224 is the ImageNet pre-training size, and *k*_0_ refers to the initial level which an RoI with *w* × *h* = 224^2^ should be assigned. We set *k_0_* to 4 as an initial state, that means we use C4 as the original feature map. Intuitively, Equation (1) indicates that if the RoI’s scale becomes larger (say, 448), it will be assigned into a higher level (say, *k* = 5). Each pyramid level has respective head to process RoI further. Faster R-CNN still share parameters among all levels. In order to build a light-weight and speedy head. Before the final classification and bounding box regression layers, we employ RoI pooling to output 7 × 7 features, and add two hidden 1024 dimensions fully-connected (fc) layers. On account of ResNets not having such fc layers, these layers will be initialized randomly and then trained.

### 2.2. Complete Intersection over Union

For anchor-based method, CIoU is a metric to evaluate the correlation between predicted boxes and ground-truth boxes. IoU is defined as

(2) $$IoU=\frac{\left|A\cap^{\;}B\right|}{\left|A\cup^{\;}B\right|}$$

where *A* and *B* represent two boxes that are calculated, respectively. In the anchor-based method, except for distinguishing positive samples and negative samples, IoU chiefly serves as a loss function for bounding boxes regression because of its scale invariance. In addition to this, IoU is also applied to select predicted boxes in Non-Maximum Suppression. However, in the case of no intersection between predicted boxes and ground-truth boxes, the IoU value is 0. That means IoU could not reflect the real distance between predicted boxes and ground-truth boxes which can offers gradient for bounding boxes regression. Although different predicted boxes have the same IoU loss value, their contact radio with ground-truth boxes may differ [23].

**Figure 4:**
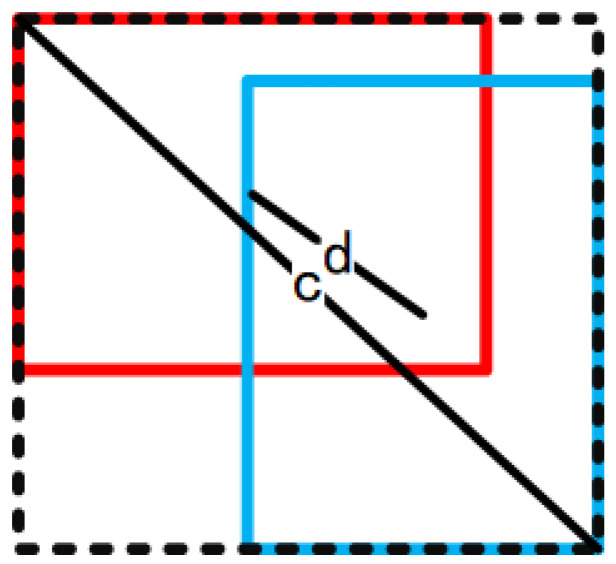
Figure of complete IoU. Red: ground-truth box, Blue: predicted box, *d*: the Euclidean distance, *c*: the minimum bounding rectangle diagonal length of predicted boxes and ground-truth boxes.

To address the above problems, CIoU, additionally takes the overlapping area, the distance between center points, and the aspect ratio of boxes into consideration, as shown in Figure 4 and CIoU’s calculation formula can be expressed as

(3) $$CIoU=1-IoU+\frac{\rho^{2}\left(b,b^{gt}\right)}{c^{2}}+\alpha v$$

where *b* refers to the center of the predicted box, *b_gt_* refers to the center of the ground-truth box, *ρ^2^* represents the Euclidean distance, *c* indicates the minimum bounding rectangle diagonal length of predicted boxes and ground-truth boxes, *α* is a weighting function and can be denoted as

(4) $$\alpha =\frac{v}{\left(1-IoU\right)+v}$$

where *v* is a metric to measure the aspect ratio consistency of predicted boxes and ground-truth boxes, calculation formula of penalty term *v* can be expressed as

(5) $$v=\frac{4}{\pi^{2}}{\left(\arctan\frac{w^{gt}}{h^{gt}}-\arctan\frac{w}{h}\right)}^{2}\;$$

where *h* and *w* refer to predicted box’s height and width, severally. *h^gt^* and *w^gt^* refer to ground-truth box’s height and width, severally.

CIoU loss includes overlapping area and the distance between center points in addition to the coverage predicted boxes. These penalty term will trigger predicted boxes to get closer with the ground-truth boxes quickly. Even in the situation that predicted boxes and ground-truth boxes have no overlap area, CIoU loss can still provide gradients for bounding boxes regression. The α weight function adds the aspect ratio penalty term to provide shape regression gradients for predicted boxes, prompting the predicted boxes to fit the size of ground-truth boxes in shape promptly. For the above reasons, CIoU can achieve better convergence speed and accuracy on the box regression problem.

### 2.3. Label Smoothing

Among multi-label classifications tasks, the softmax function is often used as the activation function of the last predicted layer to normalize the export of the network to a probability distribution over predicted classes, which can be expressed as

(6) $$p_{i}=\frac{e^{z_{i}}}{\sum_{j}e^{z_{j}}}\;$$

where *z_i_* indicates output of the last linear layer which denotes class prediction probability. Cross-entropy loss is frequently applied to evaluate the ability of a multiclassification network whose output is a probability value range from 0 to 1. Cross-entropy loss grows as the output predicted distribution *p* deviates from ground-truth label distribution *q*. The Cross-entropy loss function can be expressed as

(7) $$L=-\sum_{i}q_{i}\log p_{i}$$

where *q* is a one-hot vector that only has a sole value (1) and rest are value (0). Value (1) refers to a positive sample while value (0) refers to a negative sample. During the training process, training model get the optimal prediction probability distribution through decreasing the Cross-entropy loss between the prediction probability and ground-truth label probability. Then triggering the prediction distribution to get closer to the positive labels and away from the negative labels. Whereas, one-hot vector increases the inter-class distance between different classes and make the models become over-confident about their predictions. When training samples are so small that existing features are not enough to represent all samples’ characteristics distribution, which will drastically impair the generalization of the model and result in overfitting.

Label smoothing is a regularization method, which can be constructed for change hard label into soft label to yield robust model during training. Label smoothing can be expressed as

(8) $$q_{i}=\{\begin{array}{c} 1-\varepsilon \;\mathrm{if}\;i=y,\\ \frac{\varepsilon }{\left(K-1\right)}\;otherwise, \end{array}$$

where *K* represents the class numbers, *ε* is a constant. Label smoothing adds biases for the labels. Specifically, label smoothing regularizes a model which uses softmax with *k* classes output values by replacing the hard 0 and 1 classification targets with targets of $\frac{\varepsilon }{k-1}$ and 1−ε respectively. In other words, label smoothing decreases the class weight of ground-truth label to reduce the distance between positive samples and negative samples slightly. In this way, soft labels prevent the training models from becoming over-fitting and improves generalization of the network [24].

## 3. Experiments

The model is implemented by TensorFlow framework (version 1.4.1). TensorFlow provides libraries for building deep learning model architectures. The evaluative datasets included DeepPCB, NEU datasets, and CSU_STEEL. The FPN series networks (FPN, CFPN, and SCFPN) employ ResNet101 which pre-trained on ImageNet as backbones. During the training process, the basic learning rate is set to 0.001, and the warm-up learning rate and step learning are adopted to stabilize the initial training process. The Cross-entropy loss is introduced to measure the deviation between the predicted class and the ground-truth class. Stochastic gradient descent (SGD) minimizes the deviation and obtains the optimum weight matrix during the back propagation process. Meanwhile, the momentum algorithm is adopted to accelerate the training. Table 1 lists some hyper-parameters used in FPN series networks. All the experiments are performed on a server (12 GB NVidia Titan Xp GPU, 2.2GHz Intel Xeon E5–2630 CPU, 64GB RAM, Dell, Beijing, China).

### 3.1. Hot-Rolled Steel Strip Surface Dataset

**Figure 5:**
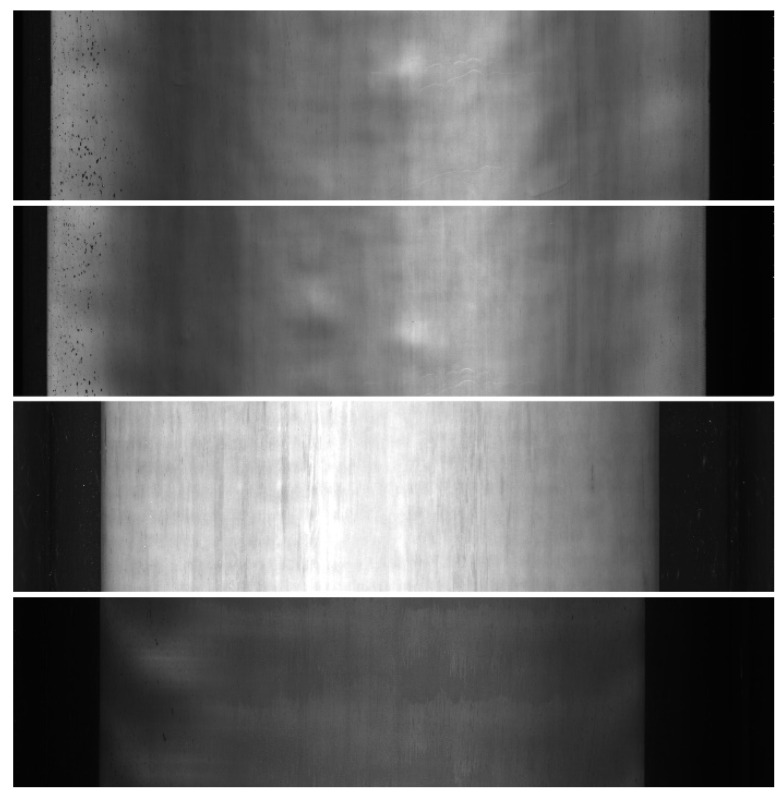
Samples of CSU_STEEL.

In the field of steel defect detection, nearly all the public datasets were processed by aforehand detection, selection, and segmentation based on prior knowledge. The samples of these datasets could not reflect the most real situation in the actual industrial production line, which has a certain impact on the performance stability of the algorithm after being transplanted to the production line. Faced with the above bottlenecks, we collected and produced a hot-rolled steel strip surface defect database called CSU_STEEL to imitate the industrial production line situation perfectly for the first time. CSU_STEEL contained 968 original images of hot-rolled steel strip surface on the industrial production line, with a size of 1024 × 4096 pixels. Figure 5 shows samples of CSU_STEEL.

CSU_STEEL has six classes of defects including roll mark, elastic deformation, wave, inclusions, oxide scale, and scratches. Different from sliced samples, each image from CSU_STEEL contains one or several classes of defects, which can be applied for both classification and detection. To our knowledge, this database is the first wide-format and high-resolution hot-rolled steel strip surface raw image dataset among peers. It provides a public dataset to verify the algorithm performance for researchers in the field of defect detection, which has contributed to the development and applications of surface defect detection field.

### 3.2. Evaluation Metrics

In our experiments, precision, recall, average precision (AP), mean average precision (mAP), and processing time are employed as evaluation metrics to investigate the performance of each network.

Precision is adapted to evaluate the percentage of correctly classified defects, and is calculated by

(9) $$precision=\frac{TP}{TP+FP}\;$$

where True Positive (*TP*) indicates the numbers that model correctly predicts the positive class, and False Positive (*FP*) indicates the numbers that model incorrectly predicts the positive class.

Recall evaluates the percentage of actual positives was identified correctly, and its calculation formula can be expressed as

(10) $$recall=\frac{TP}{TP+FN\;}\;$$

where *FN* (False Negative) refers to the numbers that model incorrectly predicts the negative class.

AP is an overall measure metric of recall and precision which is the mean of the precision after each related sample is calculated. For the sake of comprehensiveness and simplicity, AP is applied to evaluate the detection performance of a model for a certain class comprehensively.

MAP, is the average AP of each class, is adapted to evaluate the comprehensive detection performance of a model for all classes [25].

Processing time, namely the time requirement for network to process a single image, is adapted to evaluate the real-time performance of defect detection of the model.

### 3.3. Evaluation Experiments

In this paper, in order to demonstrate the performance improvement of the proposed methods, we firstly implement evaluation experiments on a widely used database, DeepPCB, which contains 1500 image pairs with annotations including positions of six common types of PCB defects [26]. With the same purpose, so as to validate the effectiveness and efficiency of the proposed methods in steel surface defect detection, especially roll mark detection. We conducted evaluation experiments on NEU and CSU_STEEL further. Each database is divided into a training set and test set by hand-out method. The methods proposed in this paper are compared with the state-of-art surface defect detection methods, including Faster R-CNN, SSD, YOLOv3, and YOLOv4. The hyper-parameters of each model are adjusted and optimized simultaneously to obtain peak performance on each dataset.

The evaluation experiments contain three parts. Primarily, in order to verify the influence of CIoU on the bounding boxes regression, the loss value comparison experiment compares the loss curves of FPN (equipped with IoU loss), CFPN (equipped with CIoU loss), and SCFPN (equipped with CIoU loss) during the training. Afterwards, the quantitative evaluation between SCFPN and other methods are executed to prove the prominent performance of proposed SCFPN. The last part is qualitative evaluation between SCFPN and other methods and the qualitative assessment will verify the validity and generalization ability of the SCFPN on hot-rolled steel strip surface roll mark detection application.

## 4. Results

### 4.1. Loss Comparision Experiment

**Figure 6:**
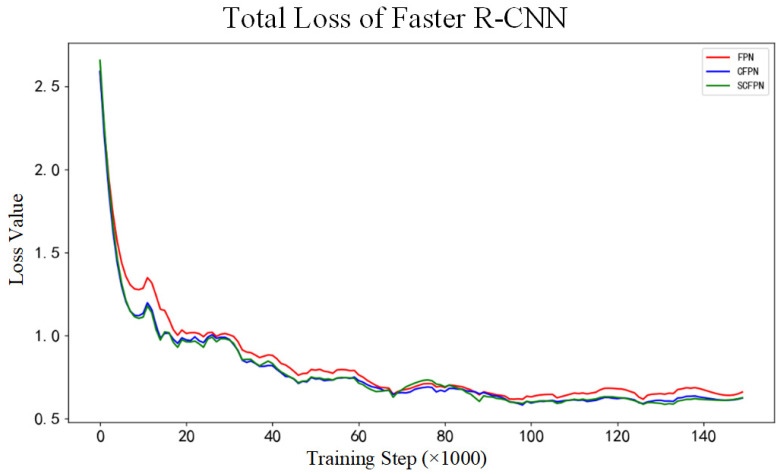
Total loss of Faster R-CNN. Red: FPN; blue: CFPN; green: SCFPN.

**Figure 7:**
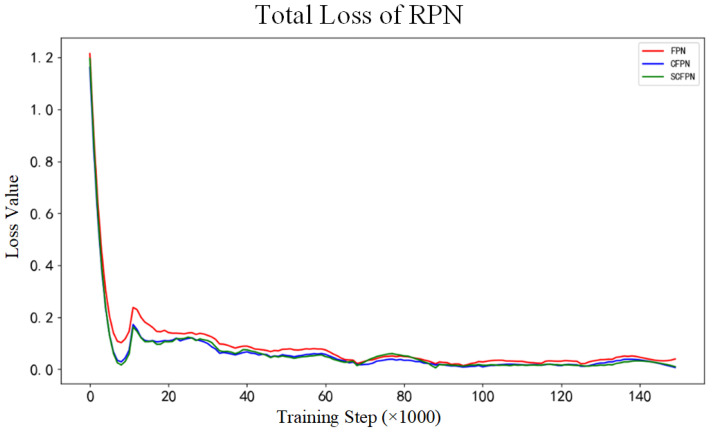
Total loss of RPN. Red: FPN; blue: CFPN; green: SCFPN.

Figure 6 shows the curves of the Faster R-CNN total loss values of each model during the training. In comparison with FPN, CFPN, and SCFPN have showed greater regression gradients in the early stage of training, and Faster R-CNN total loss of CFPN and SCFPN stabilize at 0.65 around 100,000 steps. In contrast, the Faster R-CNN total loss value of FPN still fluctuate around 0.7 after 150,000 steps. The curves of total RPN loss value of each model during the training are displayed in Figure 7. Similarly, the models of CFPN and SCFPN have greater regression gradients than FPN in the early stage of training, and smaller RPN total loss values also occur. The RPN total loss values of the three models all become stable around 0.05 at about 87,000 steps.

### 4.2. Quantitative Evaluation

AP reflects the detection performance of a model for a certain class comprehensively, and mAP is adapted to evaluate the comprehensive detection performance of a model for all classes. For the sake of a straightforward presentation, the following two experiments are only presented and analyzed by processing time, AP and mAP. Experimental evaluation results based on DeepPCB and NEU datasets are shown in Table 2 and Table 3, respectively. According to Table 2, it can be clearly known that the proposed SCFPN obtained the highest mAP, reaching 99.2%, and reached the best AP among even five types of defects. For making a fair comparison, in Table 3, we selected four more methods which have been verified on the NEU database, RAF-SSD [14], SCN [27], DIN [28], and DDN [17]. Although our SCFPN won only twice on the AP for the six distinct defect categories, it obtained the highest mAP of 82.8%.

Table 4 exhibits the roll mark detection results of each detection network on CSU_STEEL. It should be noted that Faster R-CNN and SSD are not competent to cope with the roll mark detection task of CSU_STEEL and lose detection ability. By contrast, SCFPN achieves the highest recall, accuracy and AP of 90.1%, 77.6%, and 75.9%, respectively. What is needed to demonstrate that AP of CFPN increases by 3.3% when compared with FPN, nevertheless, compared with CFPN, AP of our SCFPN further increases by 4.8%.

### 4.3. Qualitative Evaluation

**Figure 8:**
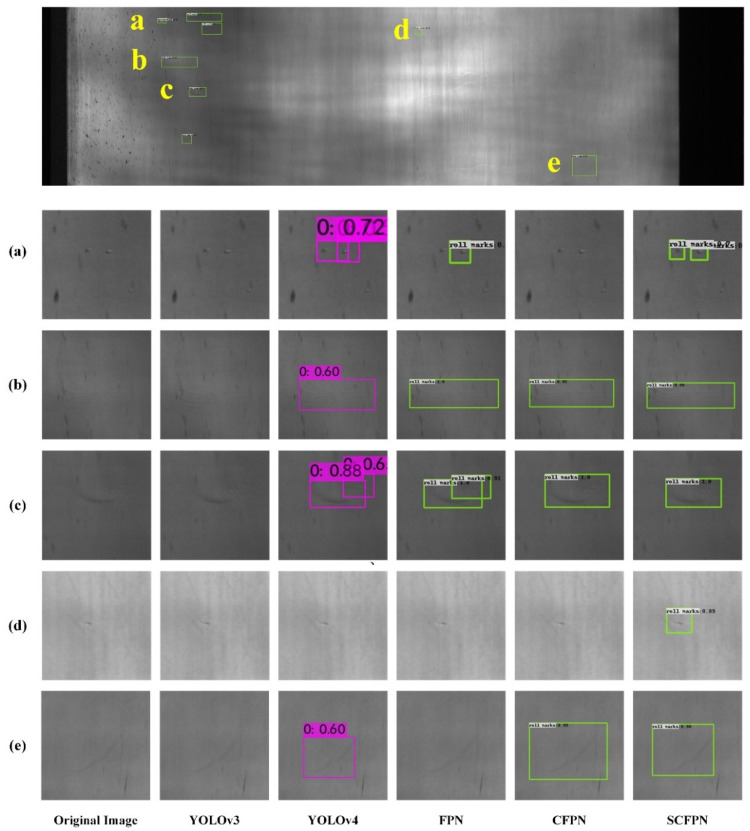
Visual detection results of different detection networks on CSU_STEEL. (**a**) tiny round roll marks; (**b**) long and narrow roll marks; (**c**) irregular roll marks with discontinuous shape; (**d**) concave roll marks with tiny line shape; (**e**) blurry and inclined roll marks

Low contrast and large intra-class distance of roll mark under inhomogeneous imaging environment bring different degrees of interference to the roll mark detection on strip steel surface. In order to verify the effectiveness of SCFPN in roll mark detection and the generalization ability confronted with multiple roll mark detection tasks, based on CSU_STEEL dataset, we select conventional and feature pyramid-based methods to conduct qualitative evaluation experiments on roll mark detection, which is shown in Figure 8. In all of our experiments based on CSU_STEEL, the original image of the hot-rolled steel strip surface is directly used for roll mark defect detection without cutting. In Figure 8, the top is the original image, and (a), (b), (c), (d), and (e) are the five kinds of high-frequency roll marks that existed in the original image, severally, and the image details are shown in the square slices below.

As can be seen from Figure 8, the roll marks can appear on any local location of the steel surface, with irregular appearance and large-scale variation, and low contrast compared to the background. For the quality control of steel strip industry, roll mark has the first alarm level, which brings great challenges to the industrial online inspection. Figure 8 shows the defect slices of the original image and the detection results of YOLOv3, YOLOv4, FPN, CFPN, and SCFPN from left to right, respectively. For type (a) roll marks, they occupy a very small area relative to the whole steel strip image and manifest in the shape of tiny round beads which are difficult to distinguish by the naked eyes in the industrial site, and usually easily submerged by tiny raindrops and rain lines in the background. Because of these characteristics, both YOLOv3 and CFPN lose their perception of such defects, FPN only detects part of these defects, our SCFPN and YOLOv4 still completely detect them. Type (b) roll marks are typical and common roll marks in the form of long and narrow trail. YOLOv3 is not sensitive to such defects, by contrast, the three methods of YOLOv4, FPN, CFPN, and SCFPN have robust cognitive ability to such defects. Type (c) roll marks are irregular roll marks with discontinuous shape, which can be detected by all other methods except YOLOv3, but FPN and YOLOv4 identifies it as two roll marks. It can also be seen from the figure that SCFPN is more accurate in identifying the location of type (c) defects. Another common roll marks are type (d) defects, which are characterized by small area proportion, tiny line shape, which are easy to be buried in noise. Only SCFPN shows excellent identification performance. Type (e) roll marks are also a common roll mark, which occupy a relatively larger area than other defects. Besides, the texture of type (e) manifests blurry, and the orientation usually inclined downward. According to the detection results, only YOLOv4, CFPN, and SCFPN can detect it.

**Figure 9:**
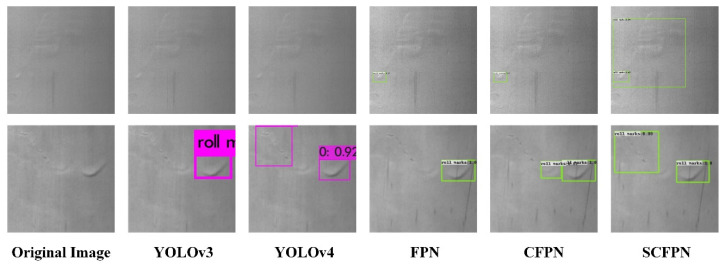
Visual detection results of different detection networks on rare roll marks.

For the comparison of detection results of rare roll marks with special morphology, as shown in Figure 9, from left to right are the original image, as well as the detection results of YOLOv3, YOLOv4, FPN, CFPN, and SCFPN. The two mentioned roll marks exhibited in the original image are rare roll marks that appear less frequently in the training dataset. For the first type of roll marks, it presents elliptic convex shape, we can observe that YOLOv3 and YOLOv4 have lost its perception ability, FPN only detects typical roll marks that occur frequently, and only part of them are detected by CFPN. In contrast, our SCFPN not only detects part of common roll marks, but also has the ability to identify rare defect forms. The second kind of roll marks are in the form of bending a broken thin line, only SCFPN and YOLOv4 can detect it with high confidence, while the other methods only detect the common roll marks and lose the identification of such defects.

## 5. Discussion

Based on the analysis of Figure 6 and Figure 7, we can draw a conclusion that CIoU endows network higher convergence speed and accuracy. In theory, the introduction of overlapping area and center distance penalty term provide the gradients about the exact direction of ground-truth boxes for the predicted boxes, even if they do not intersect. The *α* weighting function that is involved in CIoU loss function provides the gradients about shape for the predicted boxes, prompting the predicted boxes to fit the size of the ground-truth boxes in shape more quickly. Based on the above adjustments, therefore, the vanishing gradient problem resulting from IoU loss function is effectively solved. What is more, CIoU also offers better convergence speed and accuracy for bounding boxes regression.

The comparisons of experimental results of Table 2 and Table 3 indicate that CIoU loss function promotes the network in accuracy. Theoretically, α weight function in CIoU prompts the predicted boxes to fit with ground-truth boxes accurately and quickly. Label smoothing contributes to higher detection accuracy of the network ulteriorly. The intrinsic reason is that label smoothing restrains the overfitting of model and promotes the generalization ability effectively. It is worth mentioning that experimental settings and computing devices of the methods being cited are different. Therefore, the purpose of quoting them is for presentation more than comparison.

In Table 4, the proposed method achieves higher AP. However, the other methods’ performance is not very satisfactory. We can conclude that the proposed method is equipped handle a tiny target inspection task in high-resolution images captured under harsh environment. The intrinsic reason is that feature pyramid fuses multi-levels feature maps, and spreads semantic information from high layers to low layers to enrich fusing feature maps by downsampling and lateral connections. Therefore, the roll marks with large scale variation and most tiny targets can be detected stably. Contrary to methods based on multi-level feature pyramid, the networks of other methods are lack of modules which take full advantage of features, and the extracted features of these networks only contain information of respective scale which do not employ them in combination. It should be noted that YOLOv3 has feature pyramid modules, but the finest fusing feature’s resolution is 52 × 52. This results in that YOLOv3 performs poorly in tiny defect detection. In addition, the image resolution of DeepPCB and NEU datasets are 640 × 640 and 200 × 200, respectively. The scale variance between defects and the whole image is relatively small, moreover, the intra-class distance of defects is relatively small. Therefore, even methods without feature aggregation modules are competent for such detection tasks with satisfactory scores. Nonetheless, the resolution of CSU_STEEL dataset is 1024 × 4096, corresponding to the scale variance between defects and images is larger. Besides, roll marks got characteristics with low contrast and large intra-class distance, which brings about enormous difficulty for conventional object detection methods. What calls for special attention is that SCFPN is a two-stage network which implements feature extraction and regions classification and regression in two networks. YOLOv4 is a one-stage network which integrates feature extraction and object classification and regression in a single network. Nonetheless, YOLOv4 generates massive anchors result in imbalance between positive samples and negative samples. As a result, compared with our SCFPN, YOLOv4 consumes less processing time at the expense of detection accuracy. However, both methods satisfy the requirements of real-time detection of roll marks in actual steel industrial field.

By comprehensively comparing these methods in Figure 8 and Figure 9, we notice that SCFPN shows excellent recognition ability for different roll marks. Especially, for the low contrast and tiny roll marks submerged by noise, SCFPN behaves more robust. More significantly, our SCFPN is more refined for the location of defects. The comparative experiment further verifies that our proposed SCFPN has more robust feature extraction and generalization ability, which strongly adapt to the industrial application of roll mark detection in steel mills.

## 6. Conclusions

Aiming at the large intra-class distance and low contrast of roll marks of steel strips, this paper proposes a SCFPN to achieve accurate roll mark detection with high resolution image under extremely harsh industrial environments. The pyramid structure of the network possesses prominent defect feature description ability. Besides, we introduce CIoU loss function to suppress vanishing gradient during training, label smoothing is also adopted to prevent the training model become overconfident, which eases the overfitting problem. Furthermore, in view of lack of public database, we open a raw defect database of hot-rolled steel strip surface CSU_STEEL for the first time. Our method shows more robust feature extraction and fine target localization ability, performing multi-scale and fine-grained characterization to roll marks with 75.9% AP. Additionally, the SCPFN yields competitive results, with 99.2% and 82.8% mAP on DeepPCB and NEU, respectively. In addition, higher detection accuracy, stability, and generalization ability are also configured. Moreover, the proposed SCFPN belongs to end-to-end training model and is easy to implement transferring and embedding, which can provide reference for other neural networks. However, the processing time optimization and structure optimization are still in our further research.

## Figures and Tables

**Table 1:** Hyper-parameters of FPN series networks.

Hyper- Parameters	Learning Rate	Max Iteration	Momentum	Lr Decay	Batch Size	Weight Decay
Value	0.001	150,000	0.9	60,000, 120,000	4	0.0001

**Table 2:** Experimental evaluation metrics of different detection networks on DeepPCB. The bolded values are the best results in their respective columns.

Method	Processing Time/s	mAP/%	AP/%					
			Open	Short	Mousebite	Spur	Pin_Hole	Copper
Faster R-CNN	0.252	97.5	96.8	95.4	97.8	98.7	98.9	97.4
SSD	0.021	95.9	93.1	94.5	95.7	96.7	98.7	96.9
YOLOv3	**0.016**	90.9	90.9	90.8	90.9	90.7	90.9	90.9
YOLOv4	0.018	97.7	99.1	96.7	96.7	96.6	98.2	98.7
FPN	0.095	97.1	98.3	94.5	96.3	96.1	98.6	98.5
CFPN	0.094	98.9	99.2	98.8	**99.1**	98.6	99.0	98.6
SCFPN	0.091	**99.2**	**99.5**	**98.9**	98.9	**98.9**	**99.5**	**99.5**

**Table 3:** Experimental evaluation metrics of different detection networks on NEU datasets. Experimental results from methods without “*” are rerun by ourselves, and experimental results from methods with “*” are cited from published papers. The bolded values are the best results in their respective columns.

Method	Processing Time/s	mAP/%	AP/%					
			Cr	In	RS	PS	Pa	Sc
SSD	0.021	66.4	48.2	72.4	68.1	68.4	73.7	67.8
Faster R-CNN	0.122	70.4	46.2	69.7	65.4	75.2	84.6	81.5
YOLOv3	0.017	73.6	46.9	76.6	68.4	71.0	89.4	89.4
RAF-SSD *	0.019	75.1	**71.7**	75.5	75.3	72.6	80.1	75.4
SCN *	0.037	79.9	59.4	83.4	68.0	82.5	91.1	95.0
YOLOv4	**0.015**	80.4	64.2	81.8	67.8	84.6	93.0	90.9
DIN *	0.025	80.6	61.5	**85.9**	64.8	**90.3**	92.9	88.1
DDN *	0.050	82.3	62.4	84.7	**76.3**	89.7	90.7	90.1
FPN	0.089	77.3	57.3	79.5	64.8	75.9	91.6	94.8
CFPN	0.086	79.9	65.5	80.7	69.4	77.6	90.7	95.3
SCFPN	0.087	**82.8**	69.1	83.1	74.9	80.4	**93.1**	**96.4**

**Table 4:** Roll mark detection experiment evaluation metrics of different detection networks on CSU_STEEL. The bolded values are the best results in their respective columns.

Method	Recall/%	Precision/%	AP/%	Processing Time/s
Faster R-CNN	-	-	-	0.832
SSD	-	-	-	0.056
YOLOv3	22.3	63.8	18.5	**0.045**
YOLOv4	85.4	65.6	75.6	0.047
FPN	87.1	69.8	67.8	0.092
CFPN	89.4	72.7	71.1	0.091
SCFPN	**90.1**	**77.6**	**75.9**	0.091
